# Acutely elevated O-GlcNAcylation suppresses hippocampal activity by modulating both intrinsic and synaptic excitability factors

**DOI:** 10.1038/s41598-019-43017-9

**Published:** 2019-05-13

**Authors:** Hongik Hwang, Hyewhon Rhim

**Affiliations:** 1 Center for Neuroscience, Brain Science Institute, Korea Institute of Science and Technology (KIST), Seoul, 02792 Republic of Korea; 2 Division of Bio-Medical Science & Technology, KIST School, Korea University of Science and Technology, Seoul, 02792 Republic of Korea

**Keywords:** Molecular neuroscience, Neurotransmitters, Ion channels in the nervous system, Excitability, Intrinsic excitability

## Abstract

Post-translational modification (PTM) plays a critical role in increasing proteome complexity and diversifying protein functions. O-GlcNAc modification is a reversible, dynamic and highly abundant PTM catalyzed by a single pair of enzymes, O-GlcNAc transferase (OGT) and O-GlcNAcase (OGA), regardless of substrates. The two enzymes are particularly enriched in the brain, and recent proteomic studies identified that a large number of neuron-specific proteins undergo O-GlcNAc modification. In addition, pathological conditions with aberrant O-GlcNAcylation such as diabetes and obesity are associated with the higher risk of cognitive decline and memory impairment. However, despite its prevalence in the brain, functional significance of O-GlcNAcylation in regulating neuronal properties remains unclear at the molecular level. Here, we report that an acute increase in O-GlcNAcylation induced by pharmacological inhibition of OGA significantly reduces the intrinsic excitability of hippocampal CA1 neurons through the cooperative modulation of multiple voltage-gated ion channels. Moreover, elevated O-GlcNAcylation also suppresses excitatory synaptic transmission at Schaffer collateral-CA1 synapses through the removal of GluA2-containing AMPA receptors from postsynaptic densities. Collectively, our findings demonstrate that a change in O-GlcNAcylation levels dynamically regulates hippocampal activity at both intrinsic and synaptic levels, providing a mechanistic link between dysregulated O-GlcNAcylation and hippocampal dysfunction.

## Introduction

Post-translational modification (PTM) is a covalent modification of proteins that occurs following or during translation, thus contributing to diversifying protein functions. The attachment of a single O-linked β-*N*-acetylglucosamine (O-GlcNAc) moiety to proteins is one of the most common PTMs^1^. Similar to phosphorylation, O-GlcNAcylation is highly dynamic and reversible, and it only occurs on a hydroxyl group of serine and threonine residues. In this respect, phosphorylation and O-GlcNAcylation are thought to modulate each other and/or directly compete for the same modification site^2,3^, implying an important regulatory function of O-GlcNAc modification. The addition and removal of O-GlcNAc is catalyzed by a single pair of enzymes regardless of substrates, O-GlcNAc transferase (OGT) and O-GlcNAcase (OGA), respectively. OGT utilizes UDP-*N*-acetylglucosamine (UDP-GlcNAc) as the immediate donor substrate for O-GlcNAcylation which is produced when a small percentage of glucose is metabolized through the hexosamine biosynthetic pathway. The biosynthesis of UDP-GlcNAc integrates products from multiple metabolic pathways, including carbohydrate, fatty acid, and nitrogen fluxes^4^. Therefore, the primary role of O-GlcNAcylation is considered as a nutrient sensor that modulates cellular processes by reflecting the overall nutritional status within a cell^5,6^.

The identification of proteins undergoing O-GlcNAcylation and the mapping of O-GlcNAcylated sites have been challenging due to the labile nature of O-GlcNAc moieties. However, with the advances in proteomic and enrichment techniques, the number of proteins identified as O-GlcNAcylation substrates are rapidly growing^7–9^, and recent studies demonstrated the significance of O-GlcNAcylation in regulating diverse cellular functions, such as gene expression^10^, mitochondrial motility^11^, autophagy^12,13^, signal transduction^14^, neurodegeneration^15–18^. Interestingly, the list of O-GlcNAcylated proteins also includes a large number of neuronal proteins involved in neuronal development and synaptic plasticity^19–24^, such as CREB, CaMKII, and GluA2, and the expression of OGT and OGA enzymes is highly enriched in multiple brain regions^25^, including the hippocampus, cortex and hypothalamus. However, despite the prevalence of O-GlcNAc modification in the brain, the functional significance of O-GlcNAcylation in regulating neuronal functions is relatively unknown at the molecular level. Importantly, pathological conditions with abnormally high blood glucose levels such as diabetes are closely associated with cognitive deficits, dementia and neurodegeneration^26–30^, suggesting that elevated O-GlcNAcylation levels negatively influence hippocampus-dependent functions. A handful of studies examined the role of O-GlcNAcylation in regulating neuronal functions in the hippocampus, but the results from each study were not consistent in terms of synaptic plasticity and hippocampus-dependent learning^21,24,31–33^. Moreover, the use of an inhibitor with relatively low specificity such as alloxan in the preceding studies significantly hinders the interpretation of the results at the molecular level^31,32,34^. Interestingly, OGT is involved in the maturity of excitatory synapses^35^, and increasing O-GlcNAcylation levels were shown to depress epileptiform activity in the hippocampus^36^. These findings together implicate the role of O-GlcNAcylation in modulating neuronal excitability and excitatory synaptic transmission, but the underlying molecular mechanism remains elusive.

In this study, we examined how physiological properties of hippocampal neurons are affected by elevated O-GlcNAcylation levels using pharmacological inhibition of OGA, and found that acutely increasing O-GlcNAc modification strongly suppressed neuronal excitability in the hippocampus at both intrinsic and synaptic levels. In particular, elevated O-GlcNAcylation levels lowered intrinsic excitability of hippocampal CA1 neurons through concurrent and synergetic modulation of voltage-gated cation channels. In addition, elevated O-GlcNAcylation also dampened excitatory synaptic transmission at Schaffer collateral (SC)-CA1 synapses in the hippocampus by facilitating the endocytosis of GluA2-containing AMPAR receptors (AMPARs). Taken together, our results provide mechanistic insight into the modulation of hippocampal excitability via O-GlcNAcylation, and suggest a potential contribution of suppressed hippocampal activity to the cognitive deficits associated with dysregulated O-GlcNAcylation levels in the brain.

## Results

### Acutely elevated O-GlcNAcylation decreases intrinsic neuronal excitability in the hippocampus

**Figure 1:**
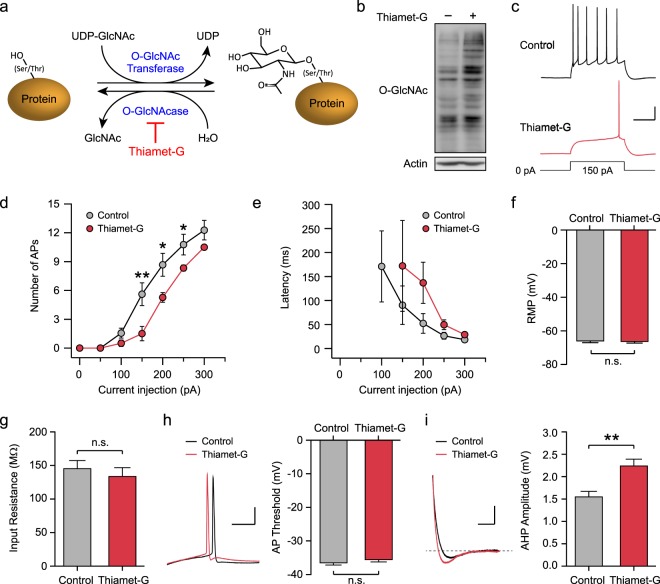
Acutely elevated O-GlcNAcylation suppresses the intrinsic neuronal excitability in the hippocampus. (**a**) Schematic diagram showing the reversible O-GlcNAc modification on serine/threonine residues of proteins mediated by O-GlcNAc transferase (OGT) and O-GlcNAcase (OGA). Thiamet-G inhibits the removal of O-GlcNAc by OGA. (**b**) OGA inhibition by thiamet-G (1 μM, 1 hr) increases overall O-GlcNAcylation levels in acute hippocampal slices. (**c**) Representative recordings of action potentials from CA1 pyramidal neurons induced by 150-pA current injection for 500 ms (scale bar: 20 mV, 200 ms). (**d**) Pharmacological inhibition of OGA decreases the number of action potentials triggered by a series of current injection (50-pA increment, 7 steps, Control: n = 9, Thiamet-G: n = 9). Each circle represents mean ± SEM (*p < 0.05, **p < 0.01, unpaired t-test). (**e**) Latency to first spike shows an increasing trend with thiamet-G treatment (Control: n = 9, Thiamet-G: n = 9). Each circle represents mean ± SEM (n.s.; not significant, unpaired t-test). (**f**) The resting membrane potential is comparable between control and thiamet-G groups (Control: n = 9, Thiamet-G: n = 9). Error bars represent ± SEM (n.s.; not significant, unpaired t-test). (**g**) The input resistance is comparable between control and thiamet-G groups (Control: n = 9, Thiamet-G: n = 9). Error bars represent ± SEM (n.s.; not significant, unpaired t-test). (**h**) Representative traces of individual action potential (left, scale bar: 20 mV, 20 ms). The threshold to initiate action potentials are not altered by elevated O-GlcNAcylation (right, Control: n = 9, Thiamet-G: n = 9). Error bars represent ± SEM (n.s.; not significant, unpaired t-test). (**i**) Representative recordings of afterhyperpolarization. A dashed line corresponds to the resting membrane potential, and the size of afterhyperpolarization was measured by the amplitude below the resting membrane potential (left, scale bar: 5 mV, 100 ms). Thiamet-G treatment increases the amplitude of afterhyperpolarization (right, Control: n = 9, Thiamet-G: n = 9). Error bars represent ± SEM (**p < 0.01, unpaired t-test). Full-length blots are presented in the Supplementary Fig. S3.

The addition of O-GlcNAc is mediated by OGT, and UDP-GlcNAc serves as an immediate donor (Fig. 1a). This modification is dynamic and reversible, and the turnover dynamics is differentially regulated among O-GlcNAcylated proteins^37^. To examine whether a change in O-GlcNAcylation levels affects physiological properties of hippocampal neurons, acute hippocampal slices were treated with thiamet-G, a selective inhibitor for OGA, for one hour. The inhibition of OGA rapidly led to a global increase in O-GlcNAcylation levels in the hippocampus (Fig. 1b). A potential change in intrinsic neuronal excitability was examined in CA1 neurons by injecting a series of 500-ms depolarization currents under current clamp in the presence of 10 μM DNQX, 50 μM D-APV and 100 μM picrotoxin. The neurons treated with thiamet-G showed a significant reduction in the number of action potential firing along with an increasing trend in the latency to first spike (Fig. 1c–e). Increasing O-GlcNAcylation by thiamet-G did not affect the resting membrane potential (RMP) (Control: −66.4 ± 0.8 mV, Thiamet-G: −66.7 ± 0.7 mV, *p* > 0.05; Fig. 1f), the input resistance (Control: 147.2 ± 11.3 MΩ, Thiamet-G: 135.6 ± 12.4 MΩ, *p* > 0.05; Fig. 1g), and the threshold for firing action potentials in CA1 neurons (Control: −36.8 ± 0.5 mV, Thiamet-G: −35.9 ± 0.5 mV, *p* > 0.05; Fig. 1h). Moreover, physiological parameters of an action potential, such as rising and decay kinetics, remained unaltered by thiamet-G (Table 1). Immediately after bursting, the membrane potential transiently drops below the RMP, a phenomenon known as afterhyperpolarization (AHP). Interestingly, thiamet-G treatment significantly increased the amplitude of AHP (Control: 1.56 ± 0.11 mV, Thiamet-G: 2.25 ± 0.14, *p* < 0.01; Fig. 1i), and the resultant hyperpolarization of the membrane potential is thought to contribute to the reduced neuronal excitability.

**Table 1:** Comparison of physiological parameters of an action potential between control and hippocampal CA1 neurons treated with thiamet-G.

	Control	Thiamet-G	*p* value
Peak amplitude (mV)	119.8 ± 1.4	122.8 ± 0.9	0.087
Time to peak (ms)	2.51 ± 0.01	2.51 ± 0.01	0.999
Half-width (ms)	1.75 ± 0.06	1.77 ± 0.04	0.774
Rise tau (ms)	0.40 ± 0.02	0.40 ± 0.01	0.803
Decay tau (ms)	1.13 ± 0.05	1.11 ± 0.04	0.835
Max rise slope (mV/ms)	237.4 ± 6.4	239.4 ± 3.0	0.777
Max decay slope (mV/ms)	−55.9 ± 1.8	−55.8 ± 1.7	0.954

The values represent the mean ± SEM (Control: n = 9, Thiamet-G: n = 9, unpaired t-test).

To further examine whether the elevated O-GlcNAcylation by thiamet-G is indeed responsible for the suppressed intrinsic neuronal excitability, we aimed to counteract the effect of thiamet-G by the co-application of OSMI-1, a cell-permeable OGT inhibitor (Supplementary Fig. S1a). Given that it takes at least two hours for OSMI-1 to significantly reduce global O-GlcNAcylation levels^38^, acute hippocampal slices were treated with vehicle, thiamet-G alone, or co-treated with thiamet-G and OSMI-1 for two hours. The co-treatment prevented the increase in overall O-GlcNAcylation levels (Supplementary Fig. S1b), and rescued the decreased excitability caused by thiamet-G alone (Supplementary Fig. S1c,d), thus suggesting that acutely elevated O-GlcNAcylation levels play a critical role in suppressing intrinsic neuronal excitability.

### Cooperative modulation of cation channels is responsible for the reduced neuronal excitability

**Figure 2:**
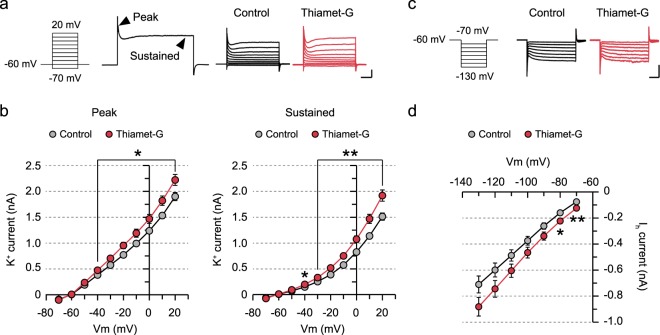
Increasing O-GlcNAcylation alters currents mediated by voltage-gated potassium channels and hyperpolarization-activated cyclic nucleotide-gated channels. (**a**) Representative recordings of potassium currents induced by a series of voltage steps (500-ms duration, 10-mV increment, 10 steps) in the presence of a mixture of channel inhibitors (200 μM CdCl_2_, 10 μM DNQX, 50 μM D-APV, 100 μM picrotoxin, 1 μM TTX). Arrows denote peak and sustained potassium currents (scale bar: 500 pA, 100 ms). (**b**) Thiamet-G treatment increases both peak and sustained potassium currents (Control: n = 12, Thiamet-G: n = 9). Each circle represents mean ± SEM (*p < 0.05, **p < 0.01, unpaired t-test). (**c**) Representative recordings of HCN-mediated currents induced by a series of hyperpolarizing voltage steps (500-ms duration, 10-mV decrement, 7 steps) in the presence of the channel inhibitor mixture (scale bar: 500 pA, 100 ms). (**d**) Thiamet-G treatment increases HCN-mediated currents under weakly hyperpolarizing conditions (Control: n = 11, Thiamet-G: n = 7). Each circle represents mean ± SEM (*p < 0.05, **p < 0.01, unpaired t-test).

Firing of action potentials is regulated by sequential and concurrent activation of multiple voltage-dependent ion channels in mammalian central neurons^39^. The activation of voltage-dependent potassium channels serves as an inhibitory driving force, and the delayed and persistent opening of voltage-dependent potassium channels after the resting potential is achieved contributes to the AHP^40–42^. Given that thiamet-G increased the amplitude of AHP and reduced intrinsic excitability, we hypothesized that an increase in the outward currents mediated by voltage-dependent potassium channels underlies the reduced intrinsic excitability observed with elevated O-GlcNAcylation. To address a compound change in the outward currents mediated by different types of potassium channels, we measured the size of the transient peak currents and sustained currents upon membrane depolarization using standard whole-cell voltage-clamp recordings. Hippocampal neurons were held at −60 mV, and subsequently given a series of voltage steps ranging from −70 to 20 mV in the presence of 200 μM CdCl_2_, 10 μM DNQX, 50 μM D-APV, 100 μM picrotoxin, and 1 μM TTX. Interestingly, acutely increasing O-GlcNAcylation significantly elevated the amplitude of both peak and sustained potassium currents in CA1 neurons (Fig. 2a,b). We then examined whether acutely increasing O-GlcNAcylation affects the cation current (I_h_) mediated by hyperpolarization-activated cyclic nucleotide-gated (HCN) channels. In the hippocampus, HCN channels are mainly present in the apical dendrites, and the I_h_ currents negatively modulate intrinsic excitability^43–48^. I_h_ current was measured by giving a series of hyperpolarizing voltage steps ranging from −70 to −130 mV, and thiamet-G treatment increased the amplitude of I_h_ currents in CA1 neurons at weakly hyperpolarized potentials (Fig. 2c,d), indicating that cation currents mediated by HCN channels contribute to the reduction in intrinsic excitability caused by pharmacological inhibition of OGA as well.

**Figure 3:**
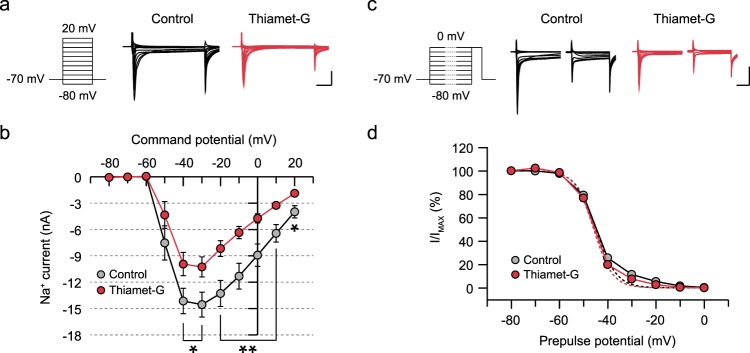
Reduced sodium influx through voltage-gated sodium channels contributes to a decrease in the intrinsic neuronal excitability caused by elevated O-GlcNAcylation. (**a**) Representative recordings of sodium currents through voltage-gated sodium channels induced by a series of voltage steps between −80 and + 20 mV (25-ms duration, 10-mV increment, 11 steps) in the presence of a mixture of channel inhibitors (200 μM CdCl_2_, 10 μM DNQX, 50 μM D-APV, 100 μM picrotoxin, 50 μM ZD7288) (scale bar: 5 nA, 5 ms). (**b**) Thiamet-G treatment strongly suppresses the amplitude of sodium currents (Control: n = 14, Thiamet-G: n = 12) at depolarized potentials. Each circle represents mean ± SEM (*p < 0.05, **p < 0.01, unpaired t-test). (**c**) Representative recordings of sodium currents induced by a series of pre-pulses (500-ms duration, 10-mV increment, 9 steps) followed by a test step (0-mV depolarization for 30 ms) in the presence of the channel inhibitor mixture (scale bar: 5 nA, 10 ms). (**d**) Peak sodium current (I) at each pre-pulse potential was normalized to the maximum peak current (I_MAX_). Steady-state inactivation curve of sodium channel was fitted by the Boltzmann equation and shown in dashed lines: I/I_MAX_ = 1/(1 + exp((V−V_1/2_)/k)) where V_1/2_ is the voltage at half-maximal availability, and k is the slope (Control: V_1/2 = _−44.79 and k = 4.244, Thiamet-G: V_1/2_ = −45.71 and k = 3.915). Availability of voltage-gated sodium channels is not affected by increasing levels of O-GlcNAc modification (Control: n = 17, Thiamet-G: n = 13). Each circle represents mean ± SEM.

Voltage-gated sodium channels also exert significant influence on the generation of action potential, and ankyrin-G serves as an anchor for voltage-gated sodium channels at the axon initial segment and nodes of Ranvier^49^. When the membrane potential is depolarized above the threshold for activating voltage-gated sodium channels, the massive influx of sodium ions mediates the initiation and propagation of action potentials along the axon. To examine the role of O-GlcNAcylation in modulating sodium influx, neurons were given a series of step depolarization ranging from −80 to 20 mV in the presence of 200 μM CdCl_2_, 10 μM DNQX, 50 μM D-APV, 100 μM picrotoxin and 50 μM ZD7288. We found that acutely elevated O-GlcNAcylation by thiamet-G remarkably attenuated the size of sodium currents in hippocampal CA1 neurons compared to the control group (Fig. 3a,b), thus contributing to the reduced intrinsic excitability. Next, we examined the influence of O-GlcNAcylation on voltage-dependent steady-state inactivation of sodium channels, given the possibility that the reduced availability of sodium channels is responsible for the decrease in sodium currents. The steady-state inactivation was measured by giving a pre-pulse depolarization step (500 ms) ranging from −80 to 0 mV followed by a test pulse to 0 mV for 30 ms. The availability of sodium channels was quantified by normalizing the size of sodium currents triggered by a test pulse at each pre-pulse potential to the current measured with −80 mV pre-pulse, and we found that the availability was not altered by thiamet-G treatment (Fig. 3c,d). Together, these results indicate that acutely increasing O-GlcNAcylation levels in the hippocampus suppresses the intrinsic neuronal excitability by modulating multiple cation channels in a cooperative manner.

### Acutely increasing O-GlcNAcylation suppresses excitatory synaptic transmission at SC-CA1 synapses in the hippocampus

**Figure 4:**
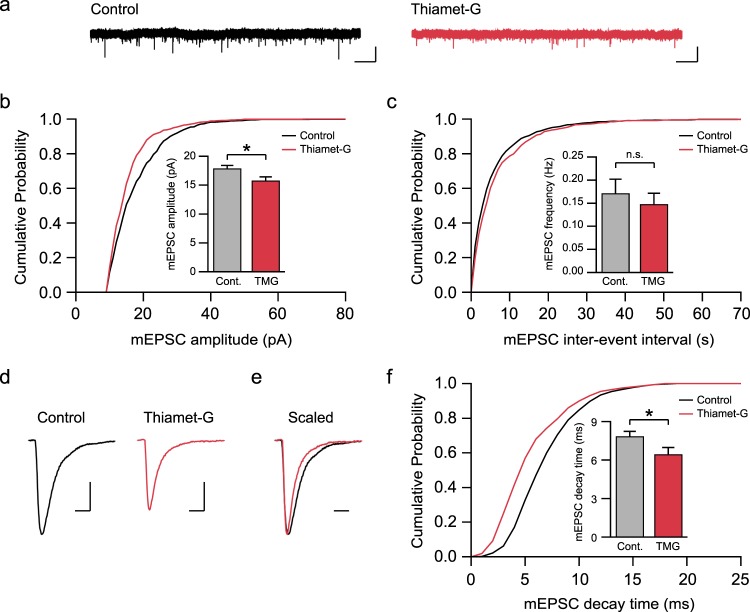
Acutely elevated O-GlcNAcylation suppresses excitatory synaptic transmission at SC-CA1 synapses in the hippocampus. (**a**) Representative recordings of AMPAR mEPSCs from control and neurons treated with thiamet-G (scale bar: 20 pA, 5 s). (**b**) Cumulative distribution function plot of AMPAR mEPSC amplitude. (Inset) Increasing O-GlcNAcylation decreases mEPSC amplitude (Control: n = 13, Thiamet-G: n = 10). Error bars represent ± SEM (*p < 0.05, unpaired t-test). (**c**) Cumulative distribution function plot of AMPAR mEPSC inter-event intervals. (Inset) mEPSC frequency is not altered by thiamet-G treatment (Control: n = 13, Thiamet-G: n = 10). Error bars represent ± SEM (n.s.; not significant, unpaired t-test). (**d**) Representative traces of averaged individual mEPSC from control and neurons treated with thiamet-G (scale bar: 5 pA, 10 ms). (**e**) Scaled individual mEPSCs to the same amplitude indicates that increasing O-GlcNAcylation accelerates the decay kinetics of mEPSCs (scale bar: 10 ms). (**f**) Cumulative distribution function plot of AMPAR mEPSC decay time. (Inset) mEPSC decay time is decreased by elevated O-GlcNAcylation (Control: n = 13, Thiamet-G: n = 9). Error bars represent ± SEM (*p < 0.05, unpaired t-test).

To initiate an action potential, the membrane potential needs to be sufficiently depolarized to active voltage-gated sodium channels in the axon initiation segment. In some type of neurons, such as pacemaker neurons, spontaneous activity causes significant depolarization of membrane potential, which enables the firing of action potentials even in the absence of synaptic transmission^50,51^. However, CA1 pyramidal neurons are not intrinsically active, and therefore, excitatory synaptic inputs are required as a major source of membrane depolarization. For this reason, we examined whether acutely elevated O-GlcNAcylation levels also influence excitatory synaptic transmission at SC-CA1 synapses in the hippocampus. To measure a potential change in the basal synaptic transmission at SC-CA1 synapses, after the treatment of hippocampal slices with either DMSO or thiamet-G for one hour, we recorded miniature excitatory postsynaptic currents (mEPSCs) in CA1 neurons in the presence of 1 μM TTX, 50 μM picrotoxin, and 50 μM D-APV (Fig. 4a). Interestingly, thiamet-G treatment significantly decreased the amplitude of mEPSCs (Control: 18.02 ± 0.47 pA, Thiamet-G: 15.95 ± 0.60 pA, *p* < 0.05; Fig. 4b,d) without altering the frequency of mEPSCs (Control: 0.17 ± 0.03 Hz, Thiamet-G: 0.15 ± 0.02 Hz, *p* > 0.05; Fig. 4c). In addition, thiamet-G treatment accelerated the decay of mEPSCs compared to control (Control: 7.90 ± 0.36 ms, Thiamet-G: 6.51 ± 0.51 ms, *p* < 0.05; Fig. 4d–f), indicating that acutely increasing O-GlcNAcylation levels suppresses excitatory synaptic transmission at SC-CA1 synapses not only by decreasing the size of AMPAR-mediated responses but also by making AMPAR-mediated currents more transient.

Based on our observation that O-GlcNAcylation levels modulate the intrinsic properties of neurons as well as excitatory synaptic transmission, we next examined whether altered O-GlcNAcylation affects basal calcium levels and ligand-induced calcium influx in hippocampal neurons. Primary hippocampal neurons grown on glass bottom dishes were loaded with Fura-2 AM for 45 min at 37 °C, and subsequently treated with either DMSO or thiamet-G for one hour. First, basal calcium levels were monitored under the resting condition in physiological saline, and thiamet-G treatment did not change basal calcium levels in soma (Control: 0.429 ± 0.004, Thiamet-G: 0.431 ± 0.006, *p* > 0.05, Supplementary Fig. S2). The hippocampal neurons were subsequently treated with a short pulse of 100 μM glutamate, which induced fast and large influx of calcium. However, the size of calcium influx in response to the glutamate pulse was comparable between control and neurons with thiamet-G treatment (Control: 1.354 ± 0.035, Thiamet-G: 1.292 ± 0.050, *p* > 0.05; Supplementary Fig. S2), which is in contrast to the decreased amplitude of mEPSCs caused by thiamet-G. This discrepancy suggests a possibility that changes in O-GlcNAcylation may differently modulate cellular processes in cultured neurons compared to acute slices. The difference may also result from the fact that the glutamate pulse activates both synaptic and extrasynaptic AMPARs, and other types of glutamate receptors in addition to AMPARs. Moreover, calcium imaging does not directly monitor sodium influx caused by the AMPAR activation, but rather it detects the influx of calcium ions, a secondary response caused by membrane depolarization. We also examined NMDA-induced calcium influx in the hippocampal neurons, and prior to the treatment with NMDA, physiological saline was replaced with the saline containing lower Mg^2+^ concentrations (30 μM MgCl_2_) and 5 μM glycine, a co-activator for NMDARs, to ensure that NMDARs can open in response to the NMDA pulse under the resting condition. Similarly to glutamate, a short pulse of 100 μM NMDA induced a significant increase in intracellular calcium levels, but the magnitude of calcium influx was not significantly different between control and thiamet-G groups (Control: 1.303 ± 0.045, Thiamet-G: 1.221 ± 0.065, *p* > 0.05; Supplementary Fig. S2), indicating that acutely increasing O-GlcNAcylation does not affect NMDAR-mediated calcium influx in hippocampal neurons.

### Increased protein O-GlcNAcylation triggers the endocytosis of GluA2-containing AMPA receptors

**Figure 5:**
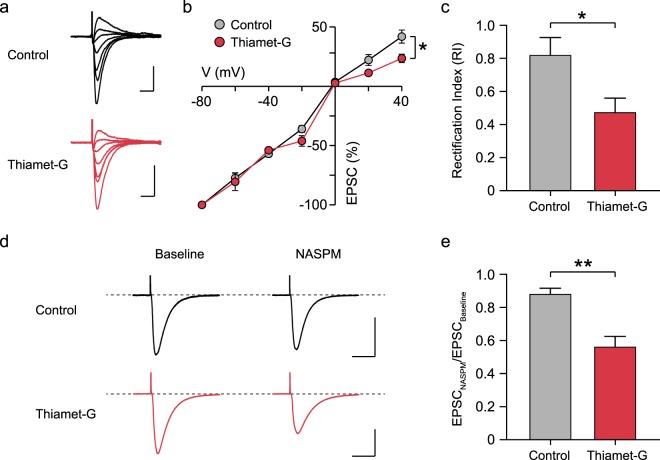
Internalization of GluA2-contaning AMPARs is responsible for the reduced excitatory synaptic transmission at SC-CA1 synapses. (**a**) Representative recording of synaptically evoked AMPAR-mediated currents at different holding potentials (scale bar: 200 pA, 20 ms). (**b**) Excitatory postsynaptic currents (EPSCs) were measured in CA1 neurons at different holding potentials ranging from −80 to + 40 mV (20-mV increment, 7 steps). The EPSC amplitudes measured at each holding potential were normalized to the EPSC amplitude measured at −80 mV holding potential. Increasing O-GlcNAcylation levels reduced the size of EPSCs at positive holding potentials (Control: n = 10, Thiamet-G: n = 7). Each circle represents mean ± SEM (*p < 0.05 at + 40 mV holding potential, unpaired t-test). (**c**) Rectification index was calculated by dividing the EPSC amplitude measured at + 40 mV holding potential to the amplitude measured at −60 mV holding potential. The ratio (+40 mV/−60 mV) was then multiplied by 1.5 to correct the difference in driving force. Thiamet-G treatment decreased the rectification index, indicating a characteristic feature of GluA2-lacking AMPARs (Control: n = 10, Thiamet-G: n = 7). Error bars represent ± SEM (*p < 0.05, unpaired t-test). (**d**) Representative recording of synaptically evoked AMPAR-mediated currents before and after NASPM treatment (scale bar: 100 pA, 40 ms). (**e**) NASPM caused significantly larger reduction in the size of EPSCs in slices treated with thiamet-G (Control: n = 6, Thiamet-G: n = 6). Error bars represent ± SEM (**p < 0.01, unpaired t-test).

A decrease in the mEPSC amplitude indicates that elevated O-GlcNAcylation induces the removal of AMPARs from synapses. AMPARs are tetramers comprising combinations of four subunits, GluA1 through GluA4, and AMPARs with different subunit composition show a distinct characteristic, such as receptor kinetics, single-channel conductance and calcium permeability^52,53^. We therefore tested whether AMPARs with a specific subunit composition are more susceptible to the internalization induced by elevated O-GlcNAcylation. Given the fact that thiamet-G treatment accelerated the decay kinetics of AMPAR-mediated currents in mEPSCs, a characteristic of GluA2-lacking AMPARs^54^, we hypothesized that increasing O-GlcNAcylation primarily induces the internalization of GluA2-contaning AMPARs. To test this hypothesis, CA1 neurons were patched under voltage clamp, and synaptically evoked currents were recorded under different holding potentials ranging from −80 to 40 mV in the presence 50 μM D-APV and 100 μM picrotoxin. Interestingly, thiamet-G treatment significantly decreased the size of AMPAR-mediated currents evoked at positive holding potentials (Control: 41.9 ± 5.5%, Thiamet-G: 23.5 ± 3.5% at + 40 mV, *p* < 0.05; Fig. 5a,b), thus making neurons more inwardly rectifying. The rectification index (RI) was calculated by dividing the size of currents measured at + 40 mV to the current measured at −60 mV, and the RI was significantly smaller in neurons treated with thiamet-G (Control: 0.81 ± 0.10, Thiamet-G: 0.47 ± 0.08, *p* < 0.05; Fig. 5c). The inwardly rectifying property is a characteristic of GluA2-lacking AMPARs, and thus acutely increasing O-GlcNAcylation is likely to weaken excitatory synaptic transmission in the hippocampus by triggering the endocytosis of GluA2-containing AMPARs. We also examined the change in the amplitude of evoked EPSCs at SC-CA1 synapses in response to NASPM, a selective channel blocker of GluA2-lacking AMPARs, and found that bath application of NASPM (100 μM, 20 min) caused significantly larger depression of EPSCs in slices treated with thiamet-G (Control: 0.88 ± 0.03, Thaimet-G: 0.56 ± 0.06, *p* < 0.01; Fig. 5d,e). Therefore, the enhanced sensitivity to NASPM as well as the inwardly rectifying property together indicate that thiamet-G treatment increases the proportion of GluA2-lacking AMPARs at SC-CA1 synapses.

## Discussion

O-GlcNAcylation is a particularly abundant PTM in the brain, and multiple neuron-specific proteins are modified by O-GlcNAc. Nonetheless, the significance of O-GlcNAcylation in regulating neuronal properties and its molecular mechanism remain elusive. In this study, we found that acutely elevated O-GlcNAcylation suppresses hippocampal activation at both intrinsic and synaptic levels. First, the intrinsic modulation involves three different voltage-gated cation channels (voltage-gated potassium channels, voltage-gated sodium channels, HCN channels), and their cooperative modulation synergistically decreased the intrinsic excitability in hippocampal CA1 neurons. Secondly, acutely elevated O-GlcNAcylation levels also suppressed excitatory synaptic transmission by decreasing the size of AMPAR-mediated currents as well as by making the currents more transient. Considering the fact that excitatory synaptic input plays a key role in depolarizing the membrane potential above the threshold for the activation of voltage-gated sodium channels in the hippocampus, elevated O-GlcNAcylation levels are expected to hinder hippocampal neurons from reaching the threshold for firing action potentials. Moreover, even when the excitatory synaptic input and the resultant depolarization is significant enough to initiate an action potential, neurons with elevated O-GlcNAcylation also suffer from a decrease in sodium influx as well as an increase in outward potassium currents. Together, these changes work in a cooperative manner and ensure that elevated O-GlcNAcylation levels suppress the activation of hippocampal CA1 neurons at multiple levels. We also previously reported that chronically elevated O-GlcNAcylation in Oga^+/−^ mice impairs synaptic plasticity without altering intrinsic neuronal excitability and basal synaptic transmission^33^, indicating that acute and chronic elevation in O-GlcNAcylation may differently modulate neuronal properties. Given the fact that O-GlcNAcylation also plays a critical role in neural development^19^, the difference may result from developmental and/or compensatory changes induced by chronically elevated O-GlcNAc modification.

Yuzwa *et al*. reported that reaching the maximum levels of cellular O-GlcNAcylation requires approximately 12 hours of exposure to thiamet-G (K_i_ = 21 nM) at 25 μM in PC12 cells^55^. Therefore, the treatment of acute hippocampal slices with thiamet-G at 1 μM for one hour is likely to trigger O-GlcNAcylation only in a subset of the O-GlcNAc target proteins. However, considering that the glucose metabolism (and thus blood glucose levels) is tightly controlled in living animals, we cautiously speculate that a condition that triggers near or complete saturation of O-GlcNAcylation is far from being physiologically relevant. Moreover, the fact that the loss of OGA in mice causes perinatal lethality further supports that a condition with saturating O-GlcNAcylation levels may quickly lead to a detrimental consequence rather than serving a modulatory function^56^. Therefore, we believe it is important to understand the role of O-GlcNAc modification under a non-saturating condition. Similarly, in a preceding study by Taylor *et al*., the authors treated hippocampal slices with thiamet-G at 1 μM only for 10 min, and were able to observe a significant increase in global O-GlcNAcylation levels along with altered synaptic transmission at CA3-CA1 synapses^21^. Besides, it would be challenging to estimate how the blockade of OGA with thiamet-G influences the degree and extend of O-GlcNAcylation levels of each individual O-GlcNAc target protein, and it is likely to vary from one substrate to another, depending on diverse factors such as subcellular localization. A study with a quantitative analysis of O-GlcNAc proteome will be able to provide valuable insights in this regard.

Unlike phosphorylation which has a specific pair of kinases and phosphatases for each substrate, O-GlcNAcylation is mediated by a single pair of enzymes, OGT and OGA, regardless of substrates. Therefore, phosphorylation caused by an external stimulus may be more suitable to regulate a specific node in a signaling pathway, but O-GlcNAc modification could be more versatile when it comes to concurrent and synergetic modulation of multiple proteins. Interestingly, recent proteomics and biochemical studies revealed that numerous neuronal proteins and ion channels are modified by O-GlcNAcylation^7,57,58^, including ankyrin-G, voltage-dependent potassium channels, small conductance calcium-activated potassium channels, voltage-gated sodium channels, voltage-gated calcium channels. In particular, ankyrin-G serves as a scaffolding protein for multiple ion channels involved in the generation and propagation of action potentials^49,59^, and its O-GlcNAcylation is known to regulate the localization of ankyrin-G at the node of Ranvier^60^, thus potentially regulating the current influx by shifting the distribution of embedded ion channels. Notably, O-GlcNAcylation of ankyrin-G was significantly down-regulated at multiple modification sites in the post-mortem human brain tissues with Alzheimer’s disease (AD)^61^, implying a potential contribution of dysregulated localization of ankyrin-G to the etiology of AD. Moreover, L254F and R284P mutations and a hemizygous T-to-G transversion in the OGT gene are associated with the higher risk of X-linked intellectual disability^62,63^. Alternatively, O-GlcNAcylation of ion channels *per se* may directly regulate current influx. For example, Ruan *et al*. reported that O-GlcNAcylation of Kcnq3 at T655 modulates the size of Kcnq3-mediated potassium currents as well as neuronal activity in AgRP neurons^64^. Voltage-gated sodium channels were also identified to undergo O-GlcNAcylation by mass spectrometry^57^, but how the channel functions are directly modulated by O-GlcNAcylation is remained to be examined.

It is also intriguing that scaffolding proteins at synapses^7,22,65^, such as Bassoon, Piccolo and Shank 2, are modified by O-GlcNAcylation, but the functional impact of their O-GlcNAcylation is currently unknown. Importantly, Tayler *et al*. reported that acutely elevating O-GlcNAcylation levels causes an NMDAR-independent form of long-term depression (LTD) at CA3-CA1 synapses in rat hippocampal slices, termed O-GlcNAc LTD^21^. They also demonstrated that the AMPAR subunit GluA2 undergoes O-GlcNAcylation and that GluA2 is required for O-GlcNAc LTD. Although our study examined SC-CA1 synapses in mouse hippocampal slices, the findings from both studies are consistent in that acutely increasing O-GlcNAcylation levels suppresses the magnitude of excitatory synaptic transmission. Moreover, our study further proved the necessity of the GluA2 subunit by demonstrating that GluA2-containing AMPARs are the primary target for endocytosis and that synapses exhibit the characteristics of GluA2-lacking AMPARs upon elevated O-GlcNAcylation levels. Stewart *et al*. also showed that epileptiform activity at CA3-CA1 synapses induced by GABA_A_R inhibition is attenuated by acutely increasing O-GlcNAclyation, and that GluA2 is required to attenuate the epileptiform activity^36^, which highlights a beneficial effect of increasing O-GlcNAcylation levels to treat seizure disorders and epilepsy. However, our results indicate that, under a non-pathological condition, acutely increasing O-GlcNAcylation levels may contribute to hippocampal dysfunction by severely impairing hippocampal activation, and thus, maintaining an adequate range of O-GlcNAcylation levels is critical to preserve normal brain functions. This view is further supported by the fact that both increasing and decreasing O-GlcNAcylation causes a deficit in synaptic plasticity and hippocampus-dependent learning^15,21,33^. Moreover, pathological conditions with aberrant O-GlcNAcylation levels such as obesity and diabetes are associated with cognitive deficits in both rodent models and human patients^26,27^. Hyperglycemia in diabetes is also considered as a key risk factor for cardiomyopathy and arrhythmias^20,66^, suggesting a possibility that elevated O-GlcNAclyation levels may negatively influence cardiac excitability in addition to neuronal excitability.

## Materials and Methods

### Animals

C57BL/6 mice and Sprague-Dawley rats (Orient Bio, Korea) were used in electrophysiology and primary neuron culture, respectively. All animals were housed under 12-hour light/dark cycle in a temperature- and humidity-controlled animal facility at the Korea Institute of Science and Technology, and *ad libitum* access to food and water was provided until the day of the experiments. All experimental protocols conformed to the Guide for the Care and Use of Laboratory Animals and were approved by the Institutional Animal Care and Use Committee of the Korea Institute of Science and Technology.

### Hippocampal slice preparation

6- to 7-week-old male C57BL/6 mice were anesthetized with halothane, and the anesthetic depth was monitored by testing the pedal withdrawal reflex. Once the lack of withdrawal reflex was confirmed, the brain was quickly isolated and the hippocampus was dissected. Hippocampal slices (300-μm thick) were prepared using a vibratome (Leica, VT1000S) in an ice-cold cutting buffer containing (in mM) 234 sucrose, 2.5 KCl, 1.25 NaH_2_PO_4_, 24 NaHCO_3_, 11 glucose, 0.5 CaCl_2_, 10 MgSO_4_, saturated with 95% O_2_ and 5% CO_2_. The slices were allowed to recover at 35 °C for one hour and subsequently maintained at room temperature in a recovery artificial cerebrospinal fluid (aCSF) solution containing (in mM) 124 NaCl, 3 KCl, 1.25 NaH_2_PO_4_, 26 NaHCO_3_, 10 glucose, 6.5 MgSO_4_, 1 CaCl_2_, saturated with 95% O_2_ and 5% CO_2_. Following the recovery, the slice was incubated with 1 μM thiamet-G for an hour to increase protein O-GlcNAcylation (or incubated with DMSO as a control) in a recording aCSF solution containing (in mM) 124 NaCl, 3 KCl, 1.25 NaH_2_PO_4_, 26 NaHCO_3_, 10 glucose, 1.3 MgSO_4_, 2.5 CaCl_2_, saturated with 95% O_2_ and 5% CO_2_ at 37 °C.

### Electrophysiology

A hippocampal slice was transferred to a submerged recording chamber and continuously perfused with the recording aCSF at room temperature throughout the experiments. To examine a change in intrinsic neuronal excitability, CA1 neurons were whole-cell patched in current clamp configuration and were given a series of current injection ranging from 0 to 300 pA at a 50-pA step. The recording aCSF was supplemented with 10 μM DNQX, 50 μM D-APV and 100 μM picrotoxin to block synaptic transmission. The internal solution contained (in mM) 130 K-gluconate, 10 KCl, 10 HEPES, 0.2 EGTA, 4 ATP-Mg, 0.5 GTP-Na_2_, 10 phosphocreatine-Na_2_ (pH = 7.25 and osmolality = 290 mOsm). Current injection triggered a firing of action potentials, and parameters such as the number of action potentials, resting membrane potential, the amplitude of afterhyperpolarization were analyzed. Input resistance and access resistance were monitored at the beginning and the end of experiments, and the data were discarded if the values change more than 20% during the recording. To measure the currents mediated by voltage-gated potassium channels, CA1 neurons were patched and held at −60 mV under voltage clamp configuration in the recording aCSF supplemented with a mixture of channel inhibitors, 200 μM CdCl_2_, 10 μM DNQX, 50 μM D-APV, 100 μM picrotoxin, and 1 μM TTX. The internal solution contained (in mM) 130 K-gluconate, 10 KCl, 10 HEPES, 0.2 EGTA, 4 ATP-Mg, 0.5 GTP-Na_2_, 10 phosphocreatine-Na_2_ (pH = 7.25 and osmolality = 290 mOsm). A voltage-step ranging from −70 to 20 mV was given for the duration of 500-ms with a 10-mV increment to activate voltage-gated potassium currents, and the size of peak and sustained potassium currents was monitored. Currents mediated by HCN channels were measured in voltage clamp mode in the recording aCSF supplemented with the channel inhibitor mixture. CA1 neurons were given a hyperpolarizing voltage-step ranging from −70 to −130 mV for the duration of 500-ms with a 10-mV increment.

Currents mediated by voltage-gated sodium channels were measured under a voltage-clamp configuration. CA1 neurons were held at −70 mV, and the recording aCSF was supplemented with 200 μM CdCl_2_, 10 μM DNQX, 50 μM D-APV, 100 μM picrotoxin and 50 μM ZD7288. The internal solution included (in mM) 20 TEA-Cl, 145 CsCl, 10 HEPES, 10 EGTA, 2 NaCl, 2 Mg-ATP (pH = 7.25 and osmolality = 290 mOsm). To activate voltage-gated sodium channels, the neurons were given a voltage-step ranging from −80 to + 20 mV for 25 ms with 10-mV increment, and the amplitude of sodium currents was monitored. To examine the availability of sodium channels, a pre-pulse ranging from −80 to 0 mV with 500-ms duration was given followed by transient depolarization (30-ms) to 0 mV. The availability was calculated by normalizing the peak current recorded at the 0-mV pulse after each pre-pulse potential to the peak current recorded after the −80 mV pre-pulse, and was fitted by the Boltzmann equation: I/I_MAX_ = 1/(1 + exp((V−V_1/2_)/k)) where V_1/2_ is the voltage at half-maximal availability, and k is the slope. For all sodium current recordings, series resistance was compensated up to 80%.

To examine the influence of altered O-GlcNAcylation on excitatory synaptic transmission, miniature excitatory postsynaptic currents (mEPSCs) were recorded after thiamet-G treatment. For recording mEPSCs, the aCSF was supplemented with 1 μM TTX, 50 μM picrotoxin and 50 μM D-APV. A whole-cell recording of CA1 neurons was made using pipettes of 3–7 MΩ resistance. The cells were held at −70 mV in voltage clamp configuration, and the internal solution contained (in mM) 125 CsMeSO_3_, 2.8 NaCl, 20 HEPES, 0.4 EGTA, 4 ATP-Mg, 0.5 GTP-Na_2_, 10 phosphocreatine-Na_2_, 5 QX314 (pH = 7.25 and osmolality = 290 mOsm). Recorded mEPSCs were analyzed with the MiniAnalysis software (Synaptosoft Inc.) using a detection threshold of 10 pA. A change in the rectification index was tested by measuring the amplitude of synaptically evoked currents at different holding potentials. CA1 neurons were held at −70 mV under a voltage-clamp configuration in the recording aCSF supplemented with 50 μM D-APV and 100 μM picrotoxin. The internal solution included (in mM) 125 CsMeSO_3_, 2.8 NaCl, 20 HEPES, 0.4 EGTA, 4 ATP-Mg, 0.5 GTP-Na_2_, 10 phosphocreatine-Na_2_, 5 QX314, 1 spermine (pH = 7.25 and osmolality = 290 mOsm). Schaffer collateral (SC) was stimulated at 0.33 Hz with a 2-contact cluster electrode (FHC, CE2C55) to evoke excitatory postsynaptic currents in CA1 neurons, and the holding potentials were varied from −80 to + 40 mV with a 20-mV interval. The rectification index was calculated by dividing the current amplitude recorded at + 40 mV to the current recorded at −60 mV, which was then multiplied by 1.5 to correct for the difference in driving force. The sensitivity to NASPM was measured by recording the baseline amplitude of synaptically evoked EPSCs for 10 min and then monitoring the remaining currents for 20 min in the presence of 100 μM NASPM. CA1 neurons were at −70 mV under a voltage-clamp configuration in the recording aCSF supplemented with 50 μM D-APV and 100 μM picrotoxin. The internal solution included (in mM) 125 CsMeSO_3_, 2.8 NaCl, 20 HEPES, 0.4 EGTA, 4 ATP-Mg, 0.5 GTP-Na_2_, 10 phosphocreatine-Na_2_, 5 QX314 (pH = 7.25 and osmolality = 290 mOsm). Schaffer collateral (SC) was stimulated at 0.1 Hz with a 2-contact cluster electrode (FHC, CE2C55) to evoke excitatory postsynaptic currents in CA1 neurons. For all electrophysiology experiments, data were collected with a MultiClamp 700B amplifier (Molecular Devices) digitized at 10 kHz with a Digidata 1550 digitizer (Molecular Devices). pClamp10 software (Molecular Devices) was used for data acquisition and analysis.

### Western blot

Acute hippocampal slices treated with 1 μM thiamet-G or DMSO vehicle in the recording aCSF for one hour at 37 °C were homogenized in a lysis buffer supplemented with protease and phosphatase inhibitors, and protein concentrations were determined by the Bradford protein assay. Laemmli sample buffer containing SDS was added to the protein samples, and boiled at 95 °C for 5 min. 20 μg proteins were loaded in each well, and separated by a 10% SDS-PAGE gel. The proteins were transferred to a PVDF membrane at 100 V for one hour, and the membrane was subsequently blocked with 5% skim milk in TBST (Tris-buffered saline, with 0.1% Tween 20) for one hour at room temperature. The membrane was then incubated with primary antibodies, anti-O-GlcNAc (Cat#: 9875 S, Cell Signaling, 1:1000) and anti-actin (Cat#: SC-8432, Santa Cruz, 1:1000), overnight at 4 °C. After being thoroughly washed with TBST, the membrane was incubated with a secondary antibody, anti-mouse IgG-HRP (Cat#: SC-2005, Santa Cruz, 1:5000), for one hour at room temperature. Protein bands were visualized and captured with the western chemiluminescent HRP substrate (WBKLS0100, Millipore) using the ImageQuant LAS 4000 system (GE Healthcare).

### Primary hippocampal neuron culture

Pups from a SD rat (E18) were used to prepare dissociated hippocampal neuron culture. The brains were separated from the pups using the needle of a 1-mL syringe, and each brain was cut into two pieces along the hemisphere. Meninges were carefully peeled off using forceps under a microscope, and hippocampi were isolated and placed in an ice-cold Hank’s balanced salt solution containing 10 mM HEPES (pH 7.3), 1x antibiotics (penicillin-streptomycin), 1 mM sodium pyruvate, 5.3 mM KCl, 0.4 mM KH_2_PO_4_, 137.9 mM NaCl, 0.3 mM Na_2_HPO_4_, 5.5 mM D-glucose. Isolated hippocampus were cut into smaller pieces and digested in a buffer containing 0.25% trypsin for 25 mins at 37 °C. The tube containing the digested hippocampus tissue was inverted every 5 min. Subsequently, plating media (Neurobasal media supplemented with Glutamax, B-27, 1x antibiotics and 5% FBS) was added to the tube to inactive trypsin. Digested hippocampus were triturated to separate single cells using a Pasteur pipette, and when the solution turns cloudy and there are no big chunk of tissues left, the cells were collected to the bottom with the centrifugation (1000 rpm, 3 min). After decanting the supernatant, the cell pellet was re-suspended in the plating media. Subsequently, the number of cells was counted using a hemocytometer. The cells were plated in a glass bottom dish (MatTek Corp., MA) at the density of 1.24 $\ast$ 10^5^ cells/mL. The glass bottom dishes were pre-coated with poly-D-lysine (0.05 mg/mL) overnight, and thoroughly washed with sterile water prior to use. Half of the media was replaced with the culture media (plating media without FBS) the day after plating, and then the half of the culture media was replaced every three to four days until the day of experiments. The culture medium was supplemented with fluorodeoxyuridine (FUdR) at DIV7 to inhibit the growth of glial cells.

### Intracellular calcium imaging

Intracellular calcium levels were measured in the somatic region of hippocampal neurons, and primary hippocampal neuron culture at DIV21 was used for Fura-2-based calcium imaging. Hippocampal neurons cultured on a glass bottom dish were loaded with 5 μM Fura-2 AM for 45 min at 37 °C in a physiological saline (in mM; 140 NaCl, 3.5 KCl, 0.4 NaH_2_PO_4_, 1.25 Na_2_HPO_4_, 2.2 CaCl_2_, 2 MgSO_4_, 10 glucose, 10 HEPES, pH 7.3) containing 0.001% pluronic F-127 (Cat #: P3000MP, Invitrogen, USA). The cells were subsequently treated with either DMSO or thiamet-G for an hour at 37 °C. After the treatment, the glass bottom dish containing the neurons was transferred to an imaging chamber attached to the stage of an inverted fluorescence microscope (IX51, Olympus), and the neurons were continuously perfused with the physiological saline at room temperature. MetaFluor software (Universal Imaging Corp.) was used for real-time imaging acquisition and analysis. The baseline calcium levels were monitored real-time, and each experiment was started only after confirming that the baseline calcium levels have been stabilized. Fluorescence images were acquired at 340 and 380 nm exposure with a computer-controlled filter changer (Lambda 10-2, Sutter Instrument) and a digital CCD video camera (C4742-95, Hamamatsu). Intracellular calcium levels were inferred from ratio images formed by a pixel-by-pixel division of images collected at 340 and 380 nm (340/380 ratio). The data point was collected every 2 sec. Baseline calcium levels were monitored for 150 sec in a physiological saline, and the cells were subsequently treated with a 30-sec pulse of 100 μM glutamate. Calcium responses to glutamate were quantified by measuring the peak 340/380 ratio minus the baseline for each neuron. The neurons were then washed for 3 min, and the physiological saline was switched to the saline with reduced Mg^2+^ (30 μM MgCl_2_) and glycine (5 μM), a co-agonist for NMDARs, to ensure the activation of NMDARs in the resting condition. The neurons were given a 30-sec pulse of 100 μM NMDA, and calcium responses to NMDA were quantified by measuring the peak 340/380 ratio minus the baseline for each neuron.

### Statistical analysis

All data are presented as mean ± SEM, with n indicating the total number of neurons studied. Statistical analysis of electrophysiology and calcium imaging data was performed with two-tailed unpaired t-test. Results were considered to be significantly different at *p < 0.05 and **p < 0.01. All data were analyzed using GraphPad Prism 7 (GraphPad Software), Clampfit 10.5 (Molecular Devices), MiniAnalysis (Synaptosoft) and/or Igor Pro 7 (WaveMetrics).

## Supplementary information

Supplementary Info
