# Supplementary material for: Acutely elevated O-GlcNAcylation suppresses hippocampal activity by modulating both intrinsic and synaptic excitability factors
Source: Sci Rep. 2019 May 13;9:7287. doi: 10.1038/s41598-019-43017-9 (PMC6514166; doi:10.1038/s41598-019-43017-9)
Supplement: Supplementary file 1 — Supplementary Info [file 41598_2019_43017_MOESM1_ESM.pdf]

Supplementary Information

Acutely elevated O-GlcNAcylation suppresses hippocampal activity by modulating both intrinsic and synaptic excitability factors

Hongik Hwang[1], Hyewhon Rhim[1, 2]

[1]Center for Neuroscience, Brain Science Institute, Korea Institute of Science and Technology (KIST), Seoul 02792, Republic of Korea

[2]Division of Bio-Medical Science & Technology, KIST School, Korea University of Science and Technology, Seoul 02792, Republic of Korea

Corresponding Author: Hyewhon Rhim

Center for Neuroscience, Brain Science Institute

Korea Institute of Science and Technology (KIST)

Seoul 02792, Republic of Korea

(Tel.) +82-2-958-5923

(Email) hrhim@kist.re.kr

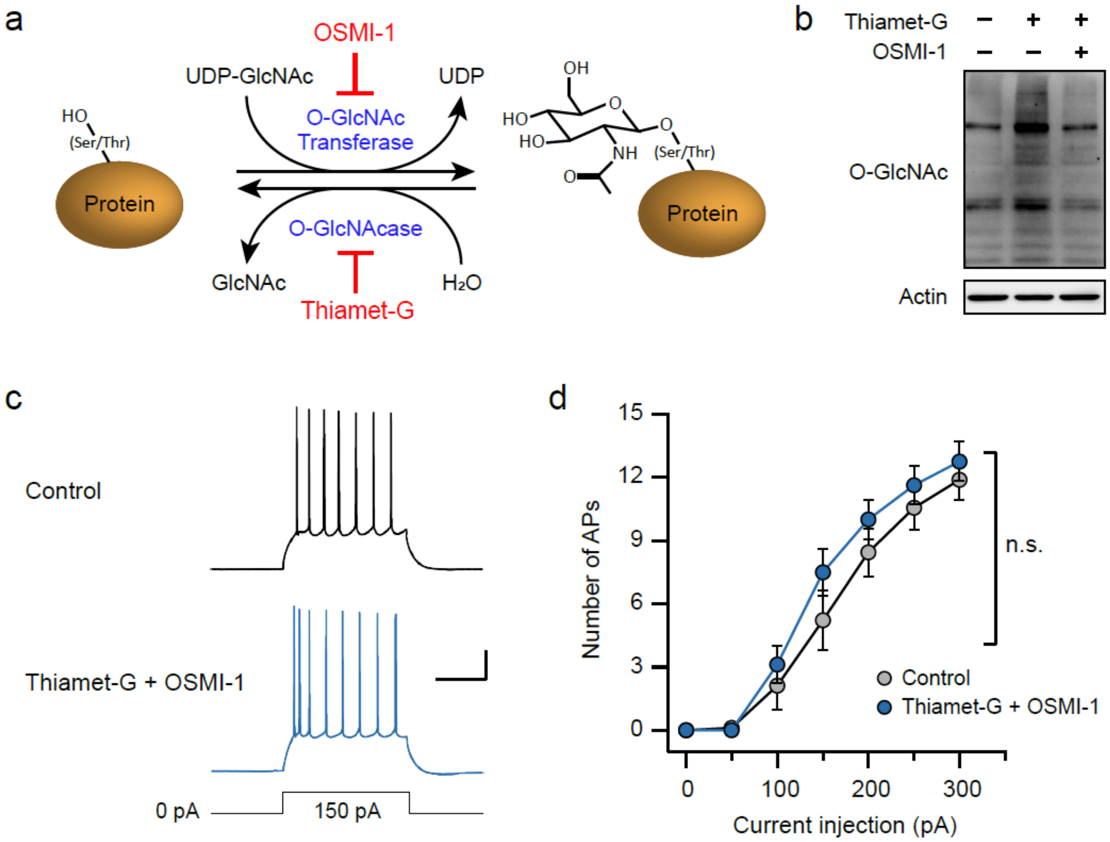

Supplementary Figure S1. Counteracting the effect of thiamet-G with an OGT inhibitor prevents a decrease in the intrinsic neuronal excitability caused by thiamet-G alone. (**a**) Schematic diagram showing the reversible O-GlcNAc modification. OSMI-1 inhibits the addition of O-GlcNAc to serine/threonine residues by OGT. (**b**) OGA inhibition by thiamet-G (1 μM, 2 hr) increases overall O-GlcNAcylation levels in acute hippocampal slices, but the co-application of an OGT inhibitor, OSMI-1 (10 μM, 2 hr), prevents the increase in O-GlcNAcylation levels. (**c**) Representative recordings of action potentials from CA1 pyramidal neurons induced by 150-pA current injection for 500 ms (scale bar: 20 mV, 200 ms). (**d**) Co-treatment of thiamet-G and OSMI-1 does not alter the number of action potentials triggered by a series of current injection (50-pA increment, 7 steps, Control: n=9, Thiamet-G: n=8). Each circle represents mean ± SEM (n.s.; not significant, unpaired t-test). Full-length blots are presented in the Supplementary Figure S4.

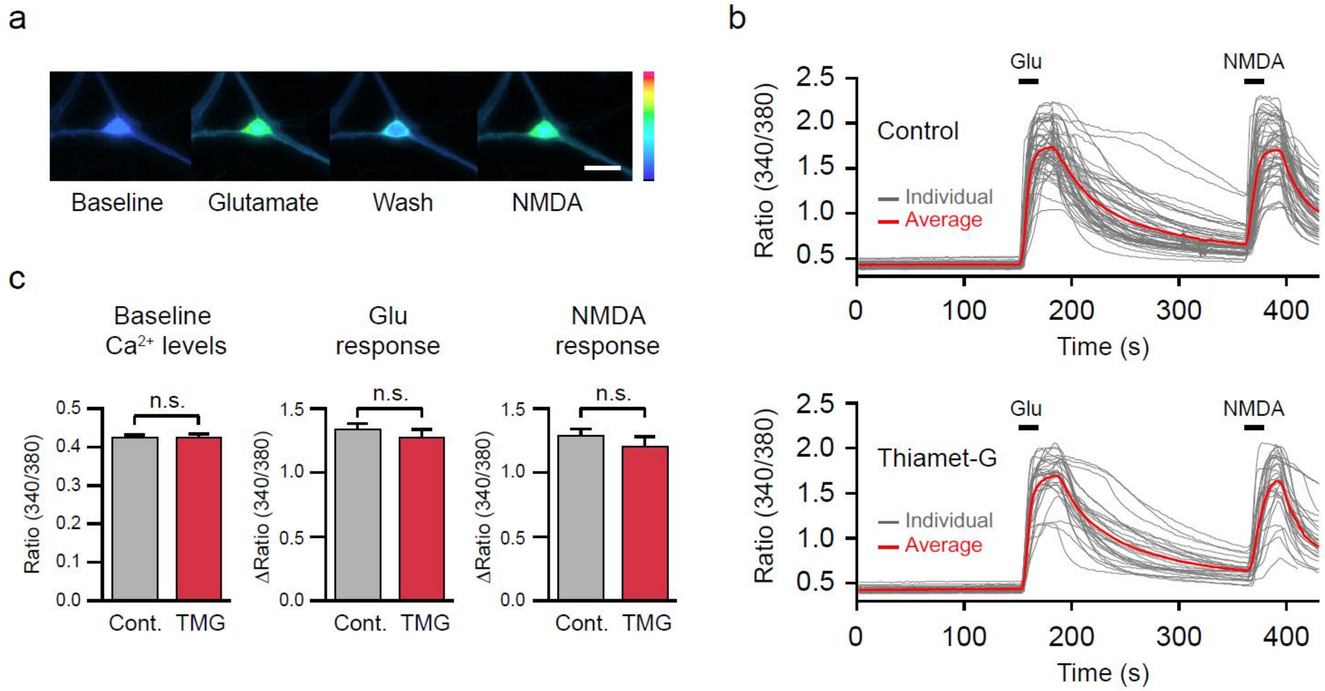

Supplementary Figure S2. Acutely elevated O-GlcNAc modification does not affect glutamate- and NMDA-induced calcium influx. (**a**) Sample images of Fura-2-based intracellular calcium levels in dissociated hippocampal neuron culture treated with a pulse of glutamate (100 μM, 30 s) or NMDA (100 μM, 30 s). Images are shown in pseudocolor reflecting the ratio of 340-nm images to 380-nm images (scale bar: 20 μm, 340/380 ratio range in pseudocolor: 0 - 3). (**b**) Glutamate and NMDA pulses triggered robust calcium influx in hippocampal neurons. Calcium responses from each individual neuron are displayed as thin gray lines, and averaged calcium responses are shown in red. (**c**) Increasing O-GlcNAcylation levels by thiamet-G did not alter resting calcium levels and the size of glutamate- and NMDA-mediated calcium influx (Control: n=51, Thiamet-G: n=27). Error bars represent ± SEM (n.s.; not significant, unpaired t-test).

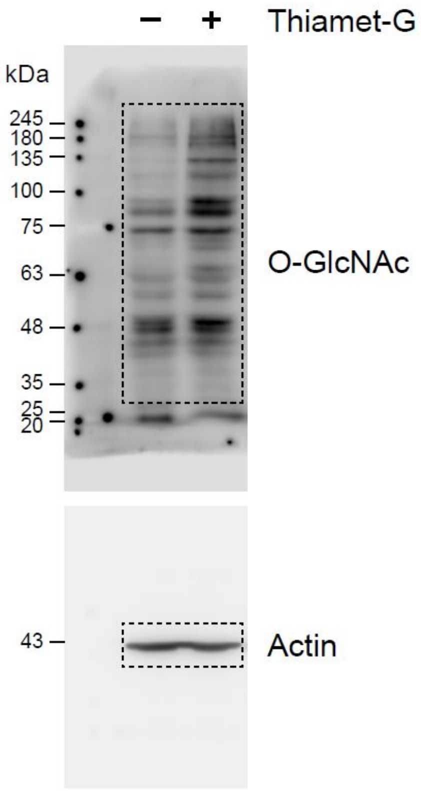

Supplementary Figure S3. Full-length blots. The original images for the blots in Figure 1b are shown. Dashed boxes indicate the cropped areas presented in the main figure.

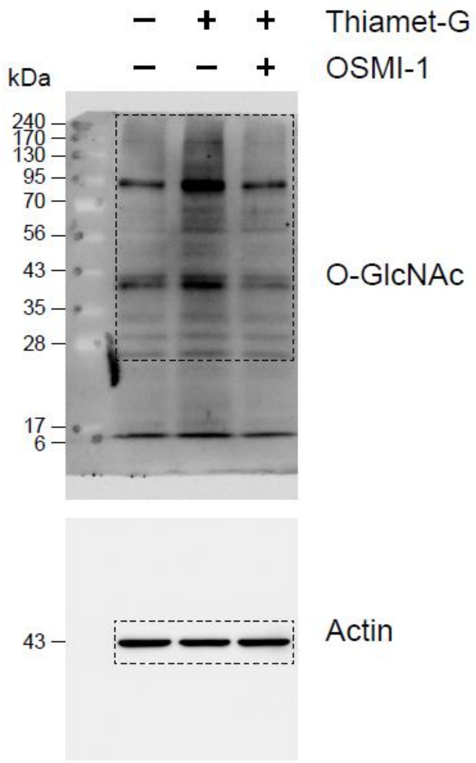

Supplementary Figure S4. Full-length blots. The original images for the blots in Supplementary Figure S1b are shown. Dashed boxes indicate the cropped areas presented in the supplementary figure.
